# Sustained activation of the AKT/mTOR and MAP kinase pathways mediate resistance to the Src inhibitor, dasatinib, in thyroid cancer

**DOI:** 10.18632/oncotarget.20488

**Published:** 2017-08-24

**Authors:** Katie M. Mishall, Thomas C. Beadnell, Brent M. Kuenzi, Dorothy M. Klimczak, Giulio Superti-Furga, Uwe Rix, Rebecca E. Schweppe

**Affiliations:** ^1^ Department of Medicine, Division of Endocrinology, Metabolism and Diabetes, School of Medicine, University of Colorado Anschutz Medical Campus, Aurora, Colorado, USA; ^2^ Department of Drug Discovery, Moffitt Cancer Center & Research Institute, Tampa, Florida, USA; ^3^ Cancer Biology PhD Program, University of South Florida, Tampa, Florida, USA; ^4^ CeMM Research Center for Molecular Medicine, Austrian Academy of Sciences, Vienna, Austria; ^5^ Center for Physiology and Pharmacology, Medical University of Vienna, Vienna, Austria

**Keywords:** Src, thyroid cancer, dasatinib, bosutinib, compound centric proteomics

## Abstract

New targeted therapies are needed for advanced thyroid cancer. Our lab has shown that Src is a key mediator of tumorigenic processes in thyroid cancer. However, single-agent Src inhibitors have had limited efficacy in solid tumors. In order to more effectively target Src in the clinic, our lab has previously generated four thyroid cancer cell lines that are resistant to dasatinib through gradual dose escalation. We further tested two additional Src inhibitors and shown the dasatinib-resistant (DasRes) cells exhibit cross-resistance to saracatinib, but are sensitive to bosutinib, suggesting that unique off-targets of bosutinib play an important role in mediating sensitivity to bosutinib. To identify the kinases targeted by dasatinib and bosutinib, we utilized an unbiased compound centric chemical proteomics screen. We identified 33 kinases that were enriched in the bosutinib pull down. Using the STRING database to map protein-protein interactions of the unique bosutinib targets, we identified a signaling axis which included mTOR, FAK, and MEK. Inhibition of the mTOR, MEK, and Src/FAK nodes simultaneously was the most effective at reducing cell growth and survival. Overall, these studies have identified key mediators of Src inhibitor resistance, and show that targeting these signaling nodes are necessary for anti-tumor efficacy.

## INTRODUCTION

Thyroid cancer is the most common endocrine cancer, with about 64,000 new cases expected to be diagnosed this year [1, 2]. The more aggressive thyroid cancers, poorly differentiated and anaplastic thyroid cancer (PDTC and ATC), typically do not respond to standard of care surgery and radioactive iodine, and patients diagnosed with anaplastic thyroid cancer (ATC) have a median survival of less than 6 months [3–5]. So far, there has been limited success treating this extremely aggressive disease, likely due to the complex molecular signatures present in these tumors [3, 6–8].

Recent genomic studies are increasing our understanding of the molecular complexity of advanced thyroid tumors. A recent study by Landa et al performed targeted sequencing (MSK-IMPACT) of 341 cancer genes, in 117 patients with advanced thyroid cancer, and identified an increase of EIF1AX, TERT and TP53 mutations; as well as an increase in overall mutations in ATC compared to PDTC [6, 9, 10]. While this information is instrumental in beginning to understand the complexity of advanced thyroid cancers, it has the potential to miss out on other key oncogenic pathways like the Src/Focal Adhesion Kinase (FAK) pathway, which are rarely mutated in cancer [11–15]. Thus, additional studies are needed to identify and investigate oncogenic pathways that are not genetically altered.

Our lab and others have previously identified Src as a key mediator of thyroid cancer cell growth, invasion, and metastasis [12–14, 16, 17]. We have also shown that inhibition of Src with two ATP competitive Src inhibitors, saracatinib and dasatinib, reduces thyroid cancer cell and tumor growth [13]. Furthermore, we have shown that these responses are due to Src activity, as introduction of the drug-resistant c-Src gatekeeper mutant abrogates response to dasatinib [13]. In addition to thyroid cancer, Src has been previously identified as an important mediator of cancer cell growth, survival, invasion, and metastasis, in a variety of cancers such as breast and lung cancer [15, 18–22]. However, despite promising pre-clinical data in thyroid and other cancers, Src inhibitors have not been as successful in solid tumor clinical trials as expected [23–25]. In order to further understand the mechanism(s) underlying this limited response; we previously generated 4 dasatinib-resistant (DasRes) cell lines, which were chronically treated with dasatinib, and investigated potential mechanisms of resistance in order to identify targets for upfront combination therapies [16]. Alongside, we generated DMSO treated control cell lines to account for any changes chronic DMSO treatment would have on the DasRes cell lines. We have successfully used this approach to identify a key role for the MAP kinase pathway, which has provided the framework for a future Src and MEK inhibitor combination clinical trial [16].

In this study, we have shown that the DasRes cells exhibit cross-resistance to the Src inhibitor, saracatinib, but interestingly are sensitive to bosutinib. We therefore investigated mechanisms mediating the sensitivity of the DasRes cell lines to bosutinib in order to identify important off-targets that may mediate dasatinib resistance. To do this we utilized a compound centric chemical proteomics approach to identify kinases that specifically bind to bosutinib. We identified that mTOR, MEK, and FAK play an important role in mediating dasatinib-resistance. Single-agent inhibition of either kinase resulted in incomplete growth inhibition, which was most likely attributed to an increase in activity of the other kinases. We further determined that inhibition of all three nodes, Src/FAK, mTOR, and MEK, results in the most effective inhibition of cell growth and increased cell death of the control and DasRes cell lines.

## RESULTS

### Dasatinib-resistant cells are cross resistant to saracatinib, but sensitive to bosutinib

**Figure 1:**
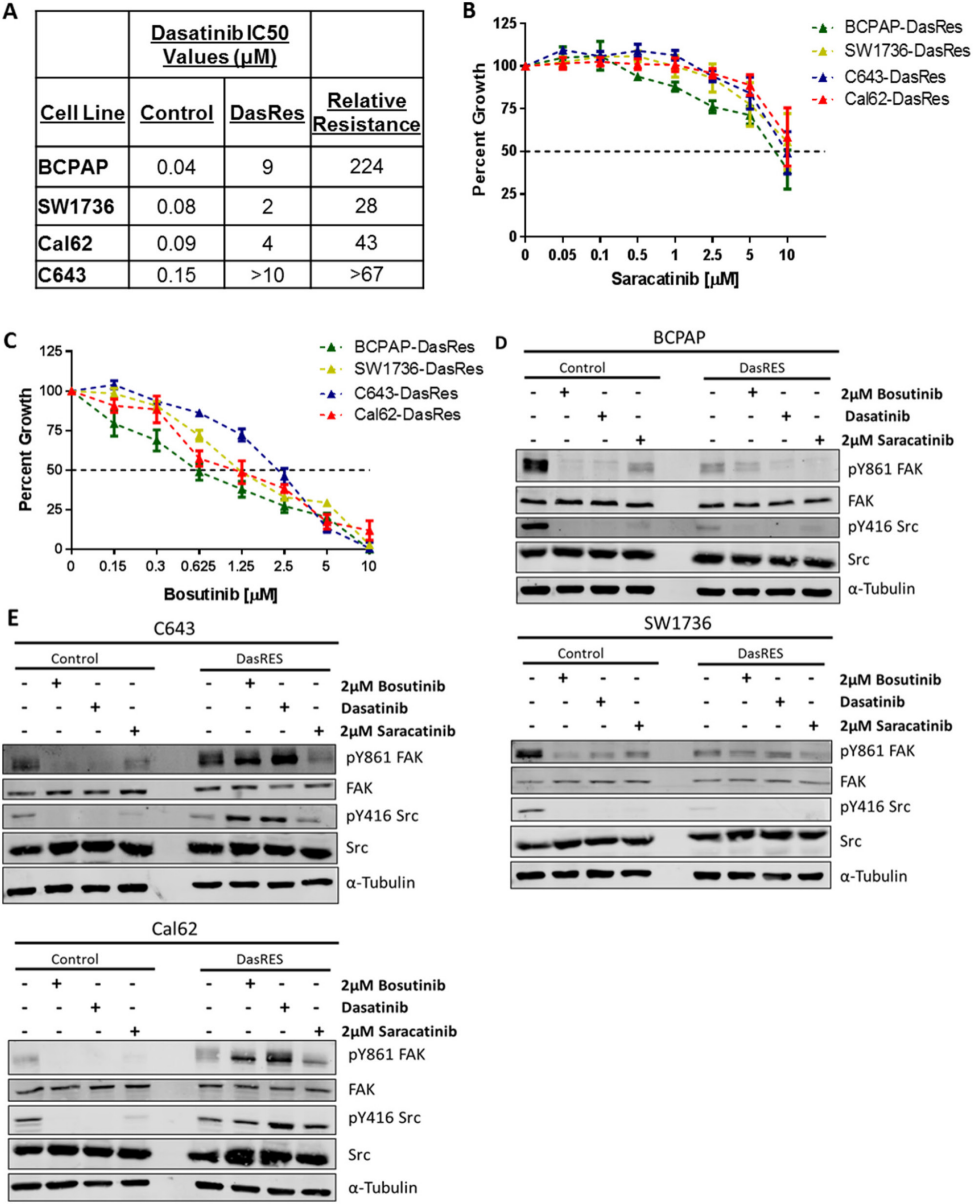
Dasatinib resistant cells exhibit differential sensitivity to the Src inhibitors, saracatinib and bosutinib **(A)** Dasatinib IC50 values of control and dasatinib-resistant (DasRes) cell lines that were calculated based on a growth curve with concentrations ranging from 0 - 10μM. **(B-C)** Sulfurhodamine B (SRB) growth assays were performed on the DasRes cell lines to determine sensitivity to saracatinib (B) and bosutinib (C). Three independent biological replicates were performed, and the standard error mean is displayed in the quantification graphs. **(D-E)** BRAF-mutant (D) and RAS-mutant (E) control and DasRes whole cell lysate was harvested after 24 hour treatments of DMSO, dasatinib, bosutinib, or saracatinib. Control cell lines were treated with 100nM dasatinib, and DasRes cell lines were treated with 2μM dasatinib. FAK/Src signaling was used to determine whether each inhibitor had similar efficacy at inhibiting Src activity. Three independent biological replicates were performed, and representative blots for signaling proteins and loading controls are shown. The pY416 Src blot was stripped and reprobed for total Src.

We previously generated four dasatinib-resistant (DasRes) cell lines by culturing cells in escalating concentrations of the Src inhibitor, dasatinib, until resistance developed, and cells were able to grow in 2 μM dasatinib (IC50 values listed in Figure 1A) [16]. Alongside, we generated DMSO treated control cell lines to account for any changes chronic DMSO treatment would have on the DasRes cell lines. For these studies, we chose two *BRAF*^*V600E*^ mutant cell lines (BCPAP; SW1736), and two *RAS*-mutant cell lines (C643, *HRAS*^*G13R*^; Cal62, *KRAS*^*G12R*^), in order to represent mutations commonly observed in thyroid cancer [3, 6, 8, 26]. Interestingly, both of the DasRes *RAS*-mutant cell lines (C643; Cal62) acquired the c-Src gatekeeper mutation, T341M, as a resistance mechanism to dasatinib, and all four of the cell lines exhibited an increased reliance on the mitogen activated protein kinase (MAPK) pathway [16].

We next asked whether the DasRes cells exhibited cross-resistance to other Src inhibitors. Using a Sulforhodamine B (SRB) growth assay, as previously described [27], we found that all four DasRes cell lines are similarly resistant to saracatinib (IC50 > 5μM), but interestingly, all four DasRes cell lines are sensitive to bosutinib compared to dasatinib (p<0.0001; two-Way ANOVA with Multiple Comparisons) (Figure 1B–1C; [16]). Accordingly, we performed clustering analysis of dasatinib, saracatinib, and bosutinib to determine drug similarity profiles, and identified that while dasatinib and saracatinib cluster together; bosutinib clusters with p38 MAPK inhibitors, SB203580 and BIRB796 (data not shown). This suggests that while bosutinib is a Src/Abl inhibitor, it has a unique profile that can be exploited to identify key targets that mediate growth and survival in the DasRes cell lines.

We next investigated whether the Src inhibitors were inhibiting Src. As expected, all three Src inhibitors effectively inhibit pY416-SRC and the Src-dependent site of FAK, pY861, in the *BRAF*-mutant control and DasRes cell lines, BCPAP; SW1736 (Figure 1D, Supplementary Figure 1A). The only exception to this is the lack of inhibition of pY861-FAK in the SW1736-DasRes cell line when treated with dasatinib (Figure 1D, Supplementary Figure 1A). We also observed over a three-fold decrease in pY416-SRC and pY861-FAK in the *RAS*-mutant control cell lines, C643; Cal62, (Figure 1E, Supplementary Figure 1B). Of note, the C643-DasRes and Cal62-DasRes cell lines acquired the c-Src gatekeeper mutation T341M, thus Src is refractory to inhibition, and interestingly, we observed a 2.5-5 fold increase in pY416-SRC and pY861-FAK in the *RAS*-mutant DasRes cell lines (C643; Cal62) with dasatinib or bosutinib, but not saracatinib treatment (Figure 1E, Supplementary Figure 1B). Interestingly, regardless of expression of the c-Src gatekeeper mutation in the *RAS*-mutant DasRes cell lines (C643; Cal62), these cell lines exhibit similar sensitivity to bosutinib as the *BRAF*-mutant DasRes cell lines (BCPAP; SW1736), suggesting a signaling dependency change that we can exploit to overcome dasatinib resistance.

### Compound centric chemical proteomics identifies bosutinib specific kinases

We hypothesized that the differential sensitivity of the DasRes cells to bosutinib is due to the ability of bosutinib to bind unique off-targets. We therefore utilized an unbiased compound centric chemical proteomics (CCCP) approach with tagged c-dasatinib and c-bosutinib to identify key targets of these drugs [28, 29]. We chose the BCPAP-DasRes cell line for this experiment, as these cells did not acquire the c-Src gatekeeper mutation, and we expected a higher level of signaling reprogramming based on our RNA-sequencing data (data not shown). Individual pulldowns were of high quality with good correlations between replicates (Supplementary Figure 2A). Using this approach, we identified a total of 75 kinases that bound to either bosutinib or dasatinib, or both (Supplementary Figure 2B, Supplementary Table 1). Importantly, identification by mass spectrometry matched well with *in vitro* kinase inhibition data for these kinases based on previous studies (Supplementary Table 2) [30–32]. Interestingly, dasatinib and bosutinib have significant differences in their drug target profiles separating them by their principal eigenvector (Supplementary Figure 2C). We therefore performed label-free quantification using Normalized Spectral Abundance Factors (NSAF) using dasatinib as a negative control bait to determine the differential drug profile of bosutinib by SAINTexpress (and vice versa) Supplementary Table 3 [33, 34].

As expected, many kinases were identified in both dasatinib and bosutinib pull down experiments and therefore denoted Dasatinib/Bosutinib Kinases, or DBKs (Supplementary Figure 2B). The DBKs provide a “proof of principle” for this approach, as many known targets of both drugs were identified, including Abl1/2, Src family kinases, and Eph family members [35]. Kinases that were predominantly identified in the dasatinib pull downs over the bosutinib pull downs are denoted as Dasatinib Specific Kinases, or DSKs, and included TGFβR1, and tyrosine-protein kinase Tec, which have both been previously identified as targets of dasatinib (Supplementary Figure 2D) [36–38].

**Figure 2:**
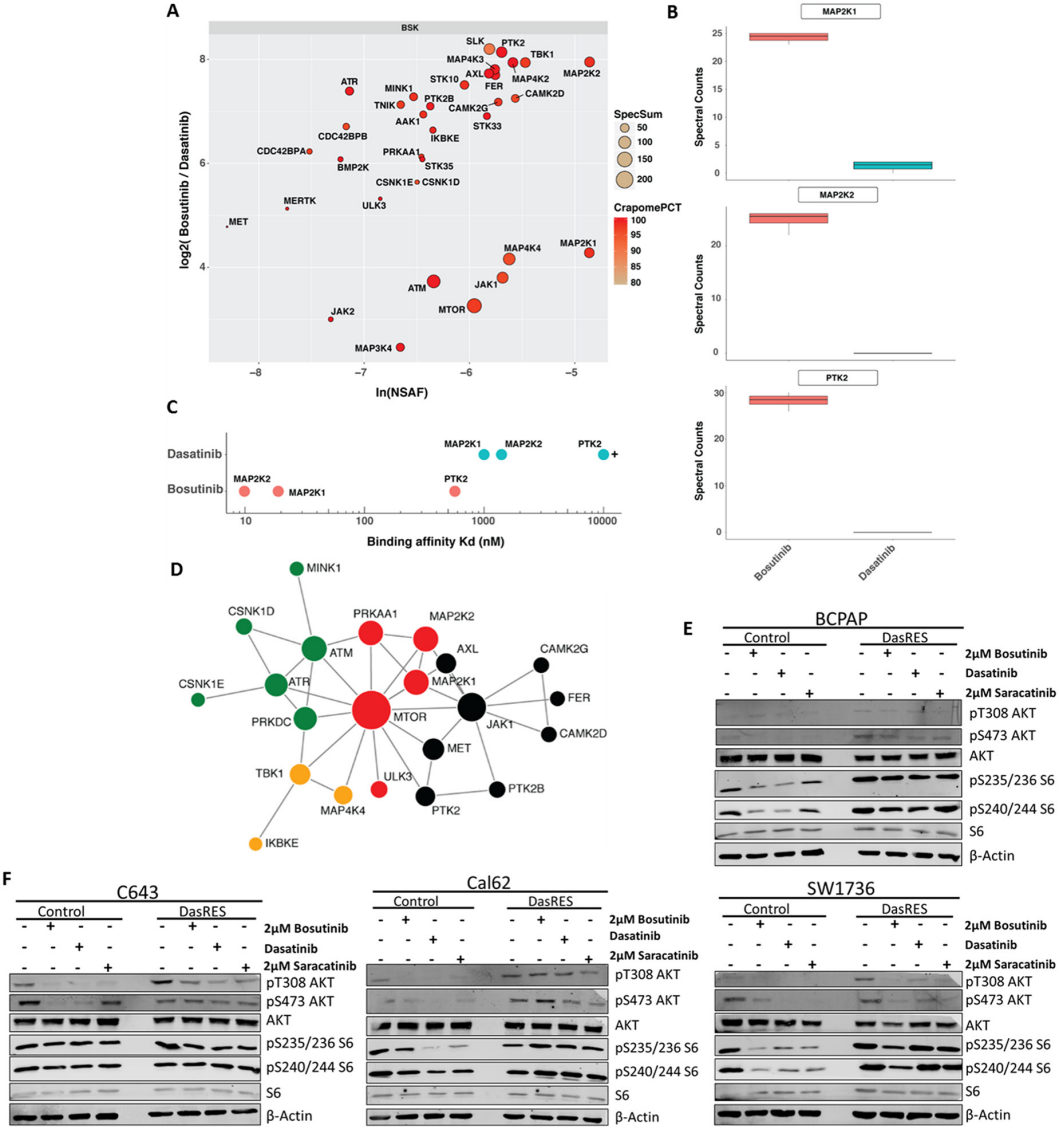
Bosutinib-specific kinase targets in BCPAP cells **(A)** Protein kinase interaction profile of bosutinib in BCPAP-DasRes cells as determined by NSAF and ratio of spectral counts relative to dasatinib. NSAF: normalized spectral abundance factor; CRAPomePCT: percent probability of specific interaction based on CRAPome database. Displayed are kinases with SaintScore >= 0.8. **(B)** Box plots of spectral counts for MEK1, MEK2 and FAK based on bosutinib and dasatinib pull downs. **(C)** Visual representation of KD‘s of relative bosutinib and dasatinib binding for MEK1, MEK2 and FAK. **(D)** STRING map of protein-protein interactions of the bosutinib specific kinases. Colors represent individual modules. Size represents eigenvector centrality. **(E-F)** BRAF-mutant (E) and Ras-mutant (F) control and DasRes cells were treated with the indicated inhibitors for 24 hours. Cell lysate was harvested and a Western blot was performed to determine changes in downstream targets of the AKT/mTOR (AKT, S6) and MEK (ERK) pathways. Three independent biological replicates were performed, and representative blots for signaling proteins and loading controls are shown. Control cells were treated with 100nM dasatinib, and DasRes cells were treated with 2μM dasatinib.

We chose to focus on the kinases identified predominantly by bosutinib pull downs, which we dubbed bosutinib-specific kinases (BSKs) (Figure 2A), as we hypothesize off-targets of bosutinib are mediating resistance to dasatinib based on our growth assay. We have previously shown that MEK1/2 (MAP2K1/2) is an important mediator of dasatinib-resistance [16]. Interestingly, MEK1 and MEK2 were both identified as some of the most prominent BSKs. Ongoing studies are defining the role of FAK (PTK2), another prominent BSK, which is also known to exhibit crosstalk with Src. MEK1/2 and FAK were prominently pulled down with bosutinib, but only minimally interacted with dasatinib (Figure 2B). This was consistent with previously reported kinase binding assays (Figure 2C) [31].

In total, over 30 kinases fell into the BSK cluster, allowing us to create a signaling map to visualize how the BSKs interact with one another. We first sought to identify an actionable signaling node that would indirectly inhibit many other kinases involved in that signaling axis. To do this, we ran the BSKs through the STRING [39, 40] database and identified JAK (enriched 14 fold over dasatinib, SaintScore = 1, EV = 0.63) and mTOR (enriched 9.5 fold over dasatinib, SaintScore = 1, EV = 1) as potential signaling nodes of relevance (Figure 2D). Interestingly, inhibition of JAK did not affect cell growth or clonogenic survival (data not shown). We therefore focused on the other key signaling node identified in the STRING analysis, mTOR, which albeit not a significant bosutinib target itself is likely interacting with one or more kinases that are direct bosutinib targets.

First, we performed Western Blotting to assess signaling of the AKT/mTOR pathway in response to Src inhibitor treatment using AKT (pT308; pS473) and S6 (pS235/236; pS240/244) phosphorylation as a readout. We observed over 50% inhibition of phospho-AKT and phospho-S6 in the *BRAF*-mutant SW1736 control cell line (BCPAP had levels too low to accurately quantify) in response to Src inhibitor treatment (Figure 2E, Supplementary Figure 2E). We also observe over 50% inhibition in the pathway in the Cal62 control cell line; however, only inhibition of AKT phosphorylation was observed in the C643 control cell line, with no change in S6 (Figure 2F, Supplementary Figure 2F). Interestingly, inhibition of the AKT/mTOR pathway was observed in two of the four DasRes cell lines: SW1736 (*BRAF*-mutant) and C643 (*RAS*-mutant); while an increase in the AKT/mTOR pathway was observed in the other two DasRes cell lines: BCPAP (*BRAF*-mutant) and Cal62 (*RAS*-mutant). We hypothesize the lack of pS6 reduction upon Src inhibitor treatment observed in the BCPAP and Cal62 DasRes cells is due to the 2 – 6 fold increase in phospho-S6 at baseline in these two DasRes cell lines compared to the respective control cell line (Supplementary Figure 2G). Accordingly, this upregulation of phospho-S6 is not observed in the SW1736- and C643-DasRes cell lines, and we do observe inhibition of this pathway in response to Src inhibitor treatment (Figure 2E–2F, Supplementary Figure 2E–2G).

### The mTOR inhibitor, everolimus, reduces thyroid cancer cell growth and inhibits S6 phosphorylation

**Figure 3:**
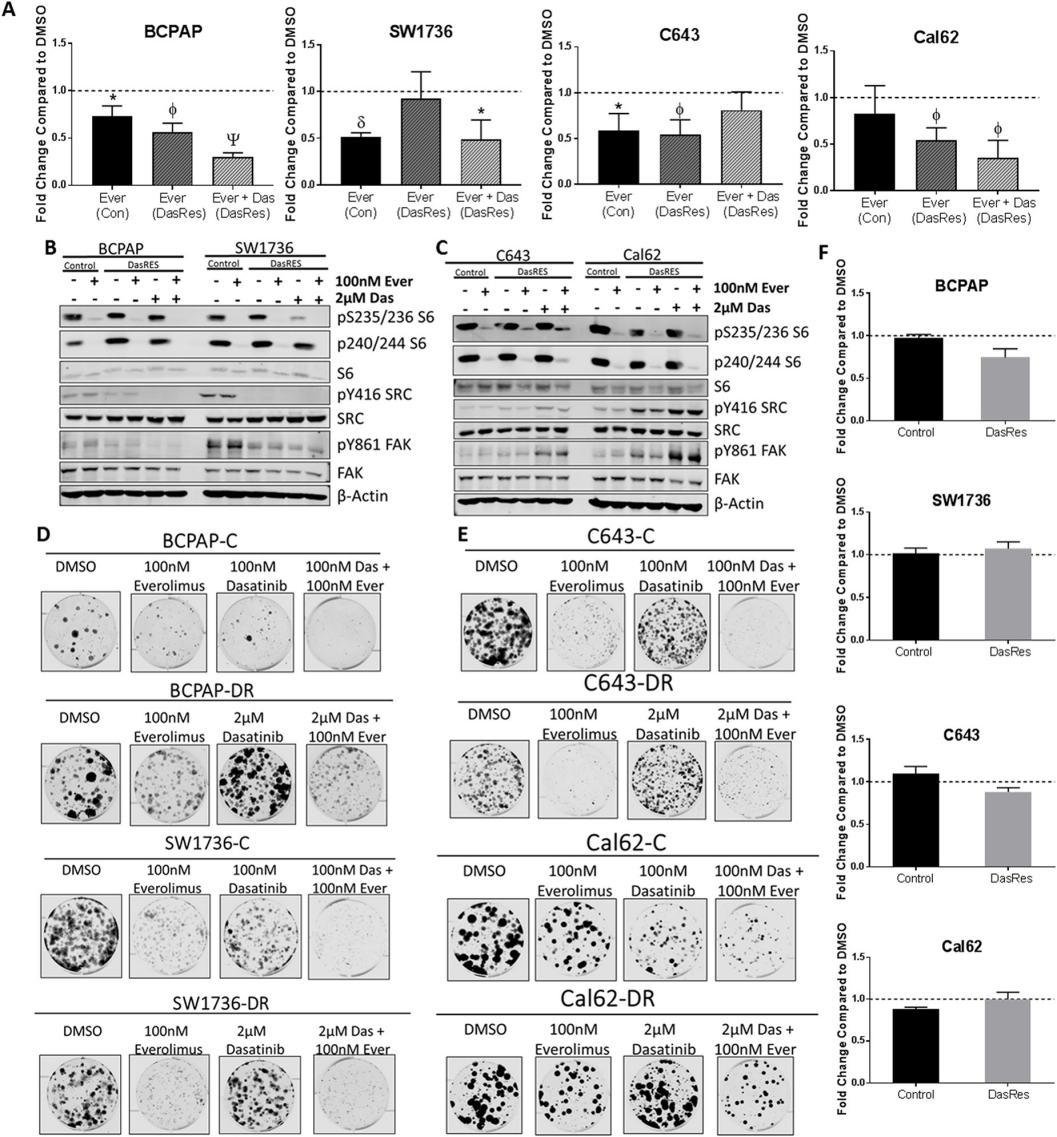
The mTOR inhibitor, everolimus, inhibits cell growth and clonogenicity, but does not induce apoptosis **(A)** Cells were treated with DMSO or Everolimus for 72 hours, and cell number was counted using the ViCell Cell Counter. Fold changes are compared to DMSO. Three independent biological replicates were performed, and the standard error mean is displayed in the quantification graphs. p-value ^*^ = 0.05 – 0.01, φ = 0.01 – 0.001, δ = 0.001 – 0.001, Ψ < 0.0001. **(B-C)** BRAF-mutant (B) and RAS-mutant (C) control and DasRes cells were treated with the indicated inhibitors for 24 hours. Cell lysate was harvested and a Western blot was performed to determine changes in the AKT/mTOR pathway signaling. Control cells were treated with 100nM dasatinib, and DasRes cells were treated with 2μM dasatinib. Three independent biological replicates were performed, and representative blots for signaling proteins and loading controls are shown. The pY416 Src blot was stripped and reprobed for total Src. **(D-E)** BRAF-mutant (D) and Ras-mutant (E) control and DasRes cells were treated with the indicated inhibitors for 7 days, and then released for 7 days to assess colony growth after inhibitor treatment. After 2 weeks, cells were fixed and stained with crystal violet. **(F)** Apoptosis was measured after 24 hours of everolimus treatment by caspase 3/7 cleavage using the Caspase-Glo 3/7 kit. Fold changes were calculated by comparing treatments to DMSO. Three independent biological replicates were performed, and the standard error mean is displayed in the quantification graphs.

In order to more specifically assess the role of mTOR in promoting DasRes growth and survival, we chose to inhibit mTORC1 with the rapalog inhibitor, everolimus [41], and assess the effect of mTORC1 inhibition on control and DasRes cell growth. For these studies, we used a Vi-CELL cell counting assay (Beckman Coulter), and treated cells with 100nM or 1μM everolimus for 3 days before assessing cell number (Figure 3A). We observed an average of 50% reduction in cell number in the everolimus treated cells compared to the DMSO treated cells. Interestingly, when the DasRes cells were maintained in 2μM dasatinib, we observed an additional 25% reduction in cell number in the *BRAF*-mutant DasRes cells (BCPAP; SW1736), but no change in the *RAS*-mutant DasRes cells (C643; Cal62) (Figure 3A). This difference is most likely due to the acquisition of the c-Src gatekeeper in the *RAS*-mutant cells, allowing Src signaling to be maintained in the presence of dasatinib.

We next evaluated signaling changes in the AKT/mTOR (S6 Ser235/236, Ser240/244) and Src/FAK (Tyr416-Src, Tyr861-FAK) pathways in response to everolimus treatment. We observed over 95% inhibition of phospho-S6 at both sites in all four control and DasRes cell lines, suggesting that mTORC1 is the primary upstream signal to activate p70S6 kinase and ultimately S6 ribosomal protein (Figure 3B–3C, Supplementary Figure 3A–3B). As expected, pY861-FAK and pY416-SRC were inhibited upon dasatinib treatment in all of *BRAF*-mutant DasRes cell lines (Figure 3B, Supplementary Figure 3A).

To determine if long term mTOR inhibition was effective at overcoming dasatinib-resistant cell growth and clonogenic survival, we performed a clonogenic experiment where we treated the control and dasatinib resistant cells with DMSO, everolimus, or everolimus plus dasatinib (Figure 3D–3E, Supplementary Figure 3C–3D). Everolimus treatment resulted in a 50% reduction in colony formation compared to vehicle. Combinatorial Src and mTOR inhibition with everolimus and dasatinib did not further decrease colony formation. Interestingly, the *BRAF*-mutant control cell lines exhibit a significant reduction in colony formation when treated with the mTOR inhibitor, everolimus. This was not necessarily expected as we hypothesized they are more reliant on MAPK signaling; however, similar growth responses have been observed in *BRAF*-mutant thyroid cancer cell lines treated with rapamycin [42].

Finally, as mTOR has been implicated as an important regulator of cell survival [43–45], we next assessed whether the reduction in cell number (Figure 3A) and colony formation (Figure 3D–3E) in response to everolimus treatment was due to cells undergoing apoptosis. We therefore performed a cleaved caspase 3/7 assay to determine whether there was an increase in apoptosis in response to everolimus treatment. Interestingly, we did not observe an induction of apoptosis after 8 hours (data not shown) or 24 hours (Figure 3F), suggesting other mechanisms such as cell growth or senescence may be involved. This is consistent with previous literature showing mTOR activity plays a role in promoting cell growth; however the functions are context dependent [46–48].

### Combinatorial inhibition of the Src/FAK, MEK, and mTOR signaling nodes is most effective at preventing dasatinib-resistant cell growth and survival

**Figure 4:**
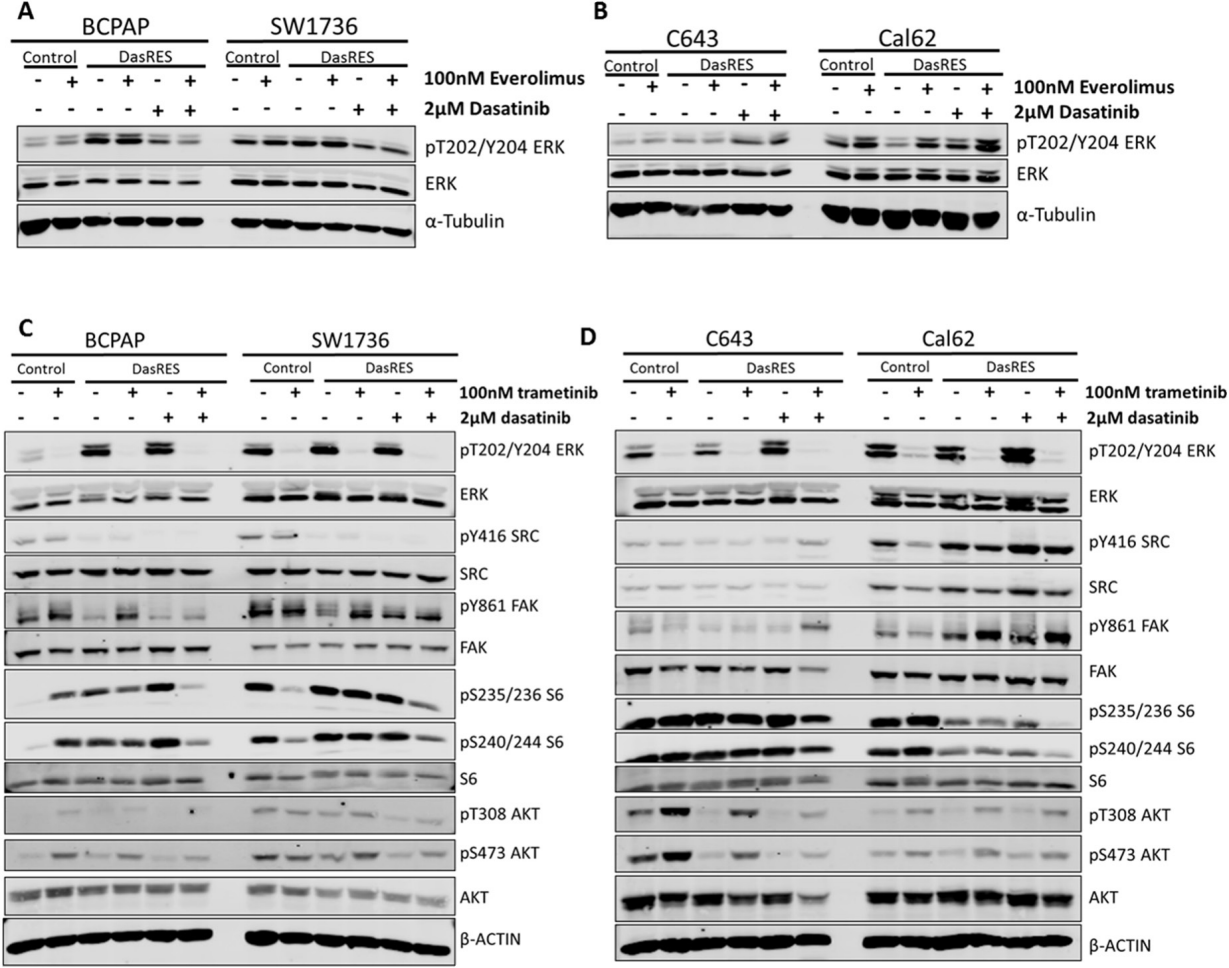
Dasatinib-resistant cells are able to switch between PI3K/AKT and MAPK pathways in response to inhibitor treatment **(A-B)** BRAF-mutant (A) and RAS-mutant (B) control and DasRes cells were treated with the indicated inhibitors for 24 hours. Cell lysate was harvested and a Western blot was performed to determine changes in MAPK pathway signaling, using phospho-ERK as a readout. **(C-D)** BRAF-mutant (C) and RAS-mutant (D) control and DasRes cells were treated with the indicated inhibitors for 24 hours. Cell lysate was harvested and a Western blot was performed to determine changes in the MAPK (ERK) and AKT/mTOR (AKT, S6) pathways. Three independent biological replicates were performed, and representative blots for signaling proteins and loading controls are shown. The pY416 Src blot was stripped and reprobed for total Src.

After only observing about 50% inhibition of growth when inhibiting mTOR alone and no induction of apoptosis, we hypothesized that other bosutinib specific kinases may be active and preventing more effective growth inhibition. We previously demonstrated that the DasRes cells are sensitive to MAPK pathway inhibition, and the combination of dasatinib and inhibition of MEK1/2 resulted in synergistic growth inhibition and induction of apoptosis [16]. Because MEK1/2 is a prominent target of bosutinib, we hypothesized that the effectiveness of bosutinib is partially due to its effects on MEK (Figure 2, Supplementary Table 2), and we therefore further explored the role MEK and mTOR are playing in DasRes cell growth and survival. Interestingly, when we treated the control and DasRes cells with everolimus, we observed a 1.5 – 4 fold increase in ppERK1/2 at sites Thr202/Tyr204 in the *RAS*-mutant cell lines (C643; Cal62), but not the *BRAF*-mutant cell lines (BCPAP; SW1736) (Figure 4A–4B, Supplementary Figure 4A–4B), suggesting that this pathway is trying to compensate for lack of AKT/mTOR activation, consistent with previous findings [49, 50].

To determine if the opposite could also occur, we first treated the control and DasRes cells with the MEK1/2 inhibitor, trametinib, to assess signaling changes in the MAPK and AKT/mTOR pathways. As expected, we observed a 95% reduction in phospho-ERK, pT202/Y204, upon trametinib treatment in the *BRAF*-mutant (BCPAP; SW1736) and *RAS*-mutant (C643; Cal62) control cell lines (Figure 4C–4D, Supplementary Figure 4C–4D), and accordingly, a reduction in pY861-FAK and pY416-SRC in the *BRAF*-mutant DasRes cell lines (BCPAP; SW1736) when maintained in dasatinib (Figure 4C, Supplementary Figure 4C). Interestingly, we observed an increase in AKT phosphorylation in three of the control and DasRes cell lines (BCPAP; C643; Cal62); suggesting the AKT/mTOR pathway is upregulated to compensate for MAPK inhibition. This has previously been observed in other tumor models [42, 51–53].

As we observe increases in either the MAPK or AKT/mTOR pathway when we inhibit only one pathway, we hypothesize that when either of these pathways is inhibited the other is upregulated to compensate. This hypothesis was based on the lack of apoptotic response and growth inhibition with mTOR inhibition alone (Figure 3). This alternative pathway upregulation and incomplete growth inhibition could lead to eventual resistance to a Src and MEK or Src and mTOR inhibitor combinations. We therefore sought to determine whether inhibition of all three nodes: Src/FAK, mTOR and MEK, was more effective than a dual combination approach.

**Figure 5:**
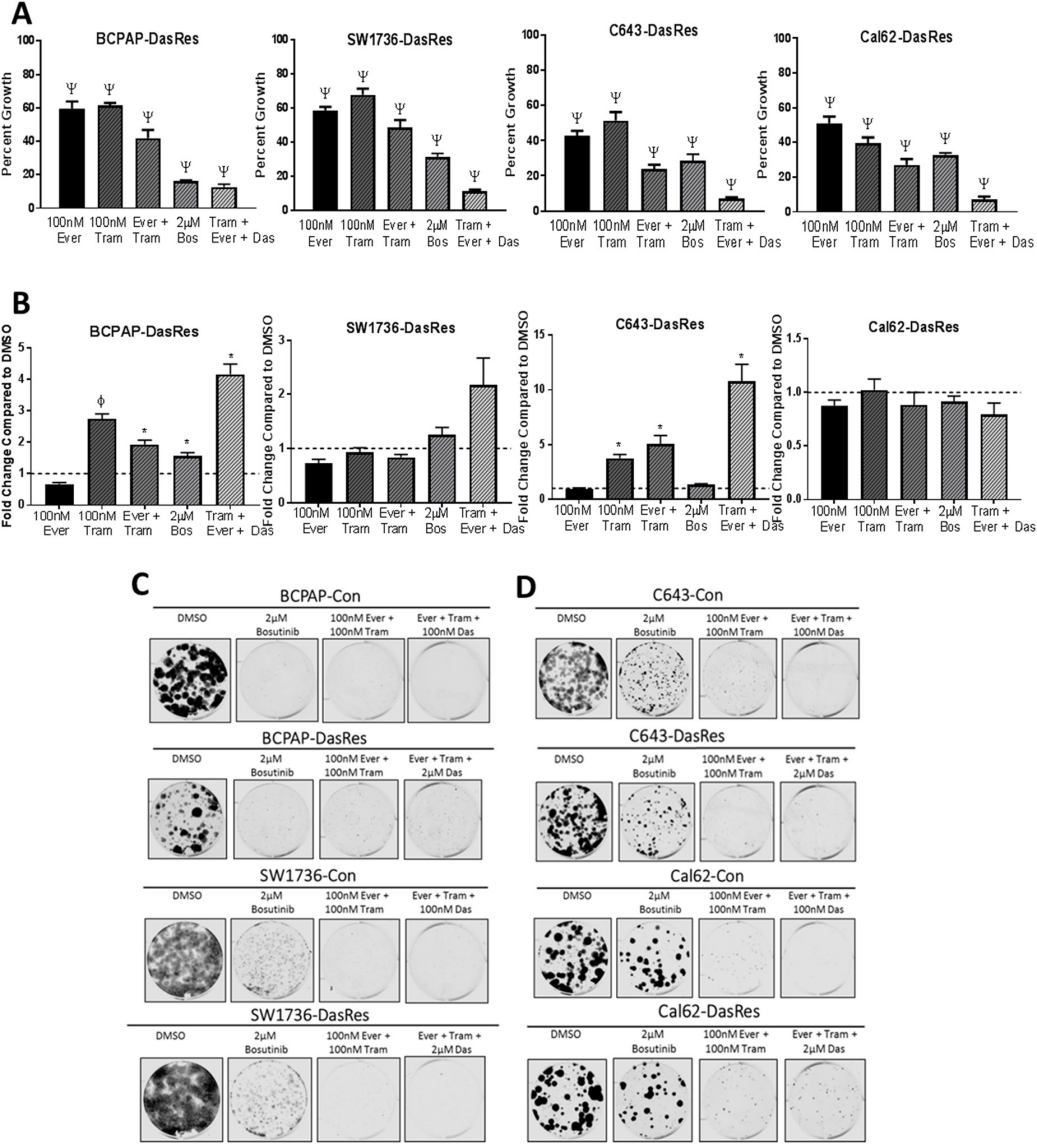
Dasatinib-resistant cells are able to utilize both PI3K/AKT and MAPK pathways to survive single agent therapy **(A)** DasRes cell growth was assessed by SRB after treatment with the indicated inhibitors for 72 hours. Three independent biological replicates were performed, and the standard error mean is displayed in the quantification graphs. p-value ^*^ = 0.05 – 0.01, φ = 0.01 – 0.001, δ = 0.001 – 0.001, Ψ < 0.0001. **(B)** Apoptosis was measured after 24 hours of treatment with the indicated inhibitors by caspase 3/7 cleavage using the Caspase-Glo 3/7 kit. Fold changes were calculated by comparing treatments to DMSO. **(C-D)** BRAF-mutant (C) and RAS-mutant (D) control and DasRes cells were treated with the indicated inhibitors for 7 days, and then released for 7 days to assess colony growth after inhibitor treatment. After 2 weeks, cells were fixed and stained with crystal violet. Fold change was calculated by comparing treatments to DMSO.

To accomplish this, we first performed a combination SRB growth assay to compare whether inhibition of these signaling nodes using bosutinib was as effective at inhibiting growth as using more potent and selective inhibitors of all three nodes with everolimus (mTOR), trametinib (MEK), and dasatinib (SRC/FAK) in the DasRes cells. Using this approach, we observed about a 50% decrease in cell growth in all four of the DasRes cell lines with either 100nM everolimus or 100nM trametinib (Figure 5A, p = <0.0001). Combination of both everolimus and trametinib treatment resulted in a 60% reduction in cell growth in all four of the DasRes cell lines (Figure 5A, p = <0.0001). We observed a similar reduction in growth (∼75%) when we treated the DasRes cells with 2μM bosutinib, further suggesting the efficacy of bosutinib is due to the inhibition of key nodes in Src/FAK, AKT/mTOR, and MAPK pathways. The greatest inhibition of growth (>85%) was observed when the DasRes cells were treated with dasatinib, everolimus, and trametinib (p = <0.0001). The triple combination was more effective than bosutinib alone; however this is to be expected, as we only observe about a 50% reduction in downstream signaling of the three nodes with bosutinib, and over 95% reduction in downstream signaling with the more potent and selective inhibitors. Furthermore, dasatinib has additional off-targets that bosutinib does not hit, thus the increased efficacy could also be due to these off-targets. Ultimately, these data suggest that inhibition of all three nodes, Src/FAK, mTOR, and MEK is the most effective approach to inhibit DasRes cell growth.

We next wanted to determine if the enhanced inhibition of cell growth observed in the SRB growth assay experiment was due to an increase in apoptosis. We have shown that everolimus does not induce apoptosis (Figure 3F), and we have previously shown that trametinib does [16]. Interestingly, combining everolimus and trametinib does not enhance apoptosis in any cell line in relation to trametinib alone, suggesting that activation of the MAPK pathway is more important in promoting cell survival than the AKT/mTOR pathway in the DasRes cell lines. Importantly, we observe the greatest induction of apoptosis when all three nodes are inhibited (Figure 5B, p = <0.05). Interestingly, bosutinib treatment did not induce apoptosis in the DasRes cell lines, suggesting that this inhibitor is more cytostatic rather than cytotoxic. As MAPK pathway inhibition induces apoptosis, this result is not too surprising, as bosutinib is not as effective as trametinib at inhibiting MEK kinase activity. The lack of bosutinib-induced apoptosis has also been observed in an *in vivo* model of thyroid cancer, in which mice treated with bosutinib exhibited a reduction in tumor growth, and this reduction was due to a decrease in key regulators of the cell cycle, including cyclins, CDK4/6, and E2F1, and not due to an increase in apoptosis [17].

We next evaluated the durability of this response using clonogenic assays with various combinations of dasatinib, everolimus, trametinib, or bosutinib. We hypothesize the effectiveness of bosutinib is due to its inhibitory effects on the Src/FAK, mTOR, and MEK signaling nodes, thus we expect that inhibiting all three of these nodes with dasatinib, everolimus, and trametinib should have a similar reduction in colony growth and survival as bosutinib, and will be more effective than either single or dual combination treatment. As expected, we observed a similar reduction in colony formation (>80%) when we treat the *BRAF*-mutant cell lines (BCPAP; SW1736) with bosutinib or the combination of everolimus and trametinib (Figure 5C, Supplementary Figure 5A). However, while the *RAS*-mutant DasRes cells (C643; Cal62) exhibit a variable 40-75% reduction in colony formation in response to bosutinib treatment, the combination treatments more effectively reduce colony formation over 90% (Figure 5D, Supplementary Figure 5B). This data further suggests the most effective approach to overcome chronic Src inhibition is to inhibit more than one key node.

## DISCUSSION

While kinase inhibitors have dramatically increased the selectivity of cancer therapeutics, it is becoming clearer that inhibiting one or two key oncogenic kinases may not be sufficient to eliminate all of the cancer cells in a tumor. Multi-kinase inhibitors, while having more off targets, may actually be more effective in combatting tumor growth, as multi-tyrosine kinase inhibitors have the potential to eliminate by-pass signaling mechanisms that can promote kinase inhibitor resistance. Selecting a drug that has the right combination of intended on- and off-targets is extremely important in preventing or delaying resistance to targeted therapies. This polypharmacology approach has previously been demonstrated to be effective in *RET*-driven thyroid cancer, using drosophila as a model to identify kinases that are contributing to tumor growth and survival [54]. Here, we show dramatic improvements in response to the co-targeting of two or three kinases simultaneously. While a triple combination therapy may have concerns regarding increased toxicity, emerging preclinical and clinical studies are showing the toxicity of triple combinations are tolerable, including a recent study in anaplastic thyroid cancer showed that a patient who did not respond to either mTOR (everolimus) or RAF/MEK (dabrafenib/trametinib) inhibitors alone, but did respond to the combination of all three kinase inhibitors [55–59].

Thyroid cancers have been particularly difficult to treat with targeted therapies, with few patients responding to MAPK pathway targeted therapies, due to a variety of resistance mechanisms, including activation of receptor tyrosine kinases, amplification of MCL1, and acquisition of the KRAS mutation in response to vemurafenib [60–63]. Recently, lenvatinib was FDA approved for radioiodine refractory thyroid cancer with little known regard to molecular mechanisms [64–66]. Through better understanding of mechanisms mediating thyroid tumorigenesis we can make appropriate inhibitor (s) selection to obtain greater responses. We have shown Src is a key player in thyroid cancer tumorigenesis and metastasis, and thus represents a clinically relevant target for advanced thyroid cancer [12–14]. Of interest, Src is upstream of the AKT/mTOR and MAPK pathways, both which have been shown to be important pathways for thyroid cancer progression [6, 8, 67, 68]. To better understand how we can improve Src inhibitor responses seen in solid tumors, we generated 4 thyroid cancer cell lines that are resistant to dasatinib to identify potential resistance mechanisms that may arise in response to chronic Src inhibition [16], in which we can target upfront to avoid the acquisition of resistance.

Interestingly, we found that the dasatinib-resistant cells were also resistant to saracatinib, but sensitive to bosutinib (Figure 1A–1C). We hypothesized that inhibition of bosutinib off-targets were responsible for the observed growth inhibition. In order to identify the kinases each inhibitor interacts with in thyroid cancer cell lines, we employed a compound centric chemical proteomics approach (Figure 2A–2C), and identified over 30 kinases that uniquely bound to bosutinib (referred to as bosutinib specific kinases, or BSKs). Using STRING analysis, we identified mTOR as a potential signaling node (Figure 2D, Supplementary Table 4). These tools allowed us to gain insight about how we could exploit key signaling nodes important in maintaining cell growth and survival in response to chronic dasatinib treatment.

Indeed, inhibition of mTOR using everolimus resulted in a 50% reduction in cell growth (Figure 3A) and over 50% reduction in colony growth (Figure 3D–3E, Supplementary Figure 3C–3D), which correlated with >90% inhibition of phospho-S6 (Figure 3B–3C, Supplementary Figure 3A–3B). Interestingly, this reduction in cell growth was not due to an increase in apoptosis, as we did not observe any increase in cleaved caspase 3/7 (Figure 3F). Taken together, we conclude that mTOR is an important promoter of control and DasRes cell growth and colony formation. Although we did not observe any induction of apoptosis, we cannot completely rule out the role mTOR plays in promoting cell death. It has previously been shown that inhibition of the AKT/mTOR and MAPK pathways can induce autophagy [69, 70]. mTOR is a negative regulator of autophagy, so inhibition of mTOR could induce autophagy, and this may be why we do not observe an induction of apoptosis in response to everolimus. However, further investigation is needed to understand the effects of mTOR inhibition on autophagy and apoptosis in thyroid cancer.

We observed an increase in the MAPK pathway in response to mTOR inhibition in the *RAS*-mutant cell lines, C643; Cal62 (Figure 4B, Supplementary Figure 4B). Consistent with previous observations in melanoma and other *RAS*-mutant cell lines, MEK inhibition with trametinib led to an increase in phospho-S6 and phospho-AKT (Figure 4C–4D, Supplementary Figure 4C–4D) [52, 71]. Accordingly, combined inhibition of the mTOR and MAPK pathways with everolimus and trametinib, respectively, resulted in enhanced inhibition of cell growth, and an increase in apoptosis (Figure 5A–5B). This reduction in growth was similar to the growth reduction observed in response to bosutinib alone (Figure 5A), suggesting partial inhibition of three key nodes with bosutinib is as effective as almost complete inhibition of two key nodes. We further observed the greatest increase in cleaved caspase 3/7 (Figure 5B) and reduction in colony growth (Figure 5C–5D, Supplementary Figure 5A–5B) when all three nodes, Src/FAK, mTOR, and MEK, are inhibited. Taken together, this data suggests that the efficacy of bosutinib is that it directly or indirectly targets all three nodes important for DasRes cell growth and survival. This is consistent with previous findings in a *RET*-driven drosophila thyroid cancer model where inhibition of key nodes: RET, RAF, Src, and S6K, was required to inhibit thyroid tumor progression [54]. Interestingly, complete inhibition of these kinases was not achieved; however a reduction in growth, invasion, and tumor volume was observed when signaling was reduced to “normal” levels.

We observe a similar trend in our model, in which even though bosutinib only modestly inhibits the downstream targets of mTOR and MEK (Figure 2E–2F, Supplementary Figure 2E–2F); it is effective at inhibiting cell growth (Figure 1C). This raised an interesting question: Is it necessary to completely inhibit protein kinase activity, or is it necessary to partially inhibit kinase activity to bring signaling down to “normal” levels? Our data would suggest that only partial inhibition of three key nodes: Src/FAK, mTOR, and MEK, is needed to prevent cell growth and increase cell death. Our data and others support the use of multi-kinase inhibitors in order to restore signaling balance in cells in many different cancer types [72–74]. However, this approach will only be effective if the inhibitor (s) targets key nodes that the cancer is dependent on for growth and survival. Thus, dissecting the molecular complexity of the tumor and identifying key nodes to disrupt these tumorigenic processes is vital for successful patient therapy.

Another interesting question arose from these data: Is Src signaling through the AKT/mTOR pathway? We do not observe a significant added benefit when we combine a Src inhibitor with an mTOR inhibitor, suggesting that maybe these proteins are signaling through the same pathway. Previous studies have shown that Src can signal through the AKT/mTOR pathway [75, 76], which is consistent with our data, as we see a decrease in phospho-AKT when we treat with all three Src inhibitors (Figure 2E–2F, Supplementary Figure 2E–2F). Future studies will investigate the role Src plays in promoting activation of the AKT/mTOR pathway, and how activation of Src increases thyroid cancer signaling plasticity. Since Src sits upstream of the AKT/mTOR and MAPK pathways, it is able to adapt to selective inhibitors targeting downstream components of each pathway. Based on these data, we hypothesize that inhibition of Src/FAK signaling, in addition to key signaling nodes such as mTOR and MEK, are vital to improve responses and prevent a therapy escape.

## MATERIALS AND METHODS

### Reagents

The c-dasatinib and c-bosutinib used for Chemical Proteomics were provided by Giulio Superti-Furga. Dasatinib and bosutinib used in the competition experiment for this screen were purchased from Chemietek and Axon Medchem, respectively. The inhibitors used for the in lab validation assays (SRB, caspase, immunoblotting) were purchased from LC Laboratories (dasatinib, bosutinib, trametinib) or SelleckChem (saracatinib, everolimus). The drugs were dissolved in dimethyl sulfoxide (DMSO) and maintained as 10mM stocks for *in vitro* studies.

### Cell culture

Human thyroid cancer cell lines BCPAP and Cal62 cells were generously provided by M. Santoro (Medical School, University of Naples Federico II, Naples, Italy), and the C643 and SW1736 cells were generously provided by Dr. K. Ain (University of Kentucky, Lexington, KY), with permission from Dr. N.E. Heldin (University Hospital, Uppsala, Sweden). Cells were grown in RPMI (Invitrogen, Carlsbad, CA) containing 5% fetal bovine serum (Hyclone Laboratories, Logan, UT) and maintained at 37°C in 5% CO_2_. All cell lines were authenticated by short tandem repeat profiling (B. Davis Center BioResources Core Facility, Molecular Biology Unit, University of Colorado) and tested for Mycoplasma contamination using the Lonza Mycoalert system (Lonza Walkersville, Inc., Walkersville, MD), according to the manufacturer’s directions. The BCPAP cell line expresses a hemizygous *BRAF*^*V600E*^ and the SW1736 cell line expresses a heterozygous *BRAF*^*V600E*^ mutation [77]. Dasatinib-Resistant cell line generation was previously described [16]. The C643 and Cal62 dasatinib-resistant cell lines acquired a heterozygous c-Src T341M mutation as previously described [16].

### Cell growth assay

Cells were plated in triplicate in 96-well plates (Sarstedt, Nümbrecht, Germany) and treated 24 hours later with increasing concentrations of the indicated inhibitors (0.05 μM to 10 μM) or DMSO (Sigma-Aldrich, United Kingdom) as a control. Cell growth was measured using Sulforhodamine B (SRB) growth assays after 72 hours post treatment. Briefly, cells were fixed with 10% trichloroacetic acid (Sigma-Aldrich) at 4°C, stained with 0.057% SRB (Sigma-Aldrich), and washed with 1% acetic acid to remove unbound stain. The protein-bound SRB was solubilized using 10 mmol/L of unbuffered Tris base, and the optical density was measured at 570nm using a SynergyH1 hybrid plate reader (Biotek, Winooski, VT). Cell growth was calculated by comparing the intensity of the stain to the DMSO control, which was set to 100%. IC50 values were calculated using nonlinear regression analysis with the GraphPad Prism software (GraphPad Software, La Jolla, CA).

### Compound centric chemical proteomics

BCPAP-DasRes cells were released from dasatinib treatment for 2 weeks and then cells were harvested and pelleted using centrifugation. Drug affinity chromatography experiments were conducted essentially as described previously [28, 29, 78]. Briefly, c-bosutinib or c-dasatinib were immobilized on NHS-activated Sepharose for Fast Flow resin (GE Healthcare) and blocked with ethanolamine overnight. BCPAP-DasRes cells were lysed and total cell lysate containing 1 mg of protein were added to the affinity matrix for 2 hours. Competition experiments were conducted by incubating total cell lysates with 20 μM bosutinib or dasatinib during affinity chromatography. Peptides were prepared by SDS-PAGE and trypsin digest as described previously [79]. A nanoflow ultra high performance liquid chromatograph (RSLC, Dionex, Sunnyvale, CA) coupled to an electrospray bench top orbitrap mass spectrometer (Q-Exactive plus, Thermo, San Jose, CA) was used for tandem mass spectrometry. The sample was first loaded onto a pre-column (2 cm x 100 μm ID packed with C18 reversed-phase resin, 5 μm, 100 Å) and washed for 8 minutes with aqueous 2% acetonitrile and 0.04% trifluoroacetic acid. The trapped peptides were eluted onto the analytical column, (C18, 75 μm ID x 50 cm, 2 μm particle size, 100 Å pore size, Dionex, Sunnyvale, CA). The 129 minute gradient was programmed as: 95% solvent A (2% acetonitrile + 0.1% formic acid) for 8 minutes, solvent B (90% acetonitrile + 0.1% formic acid) from 5% to 50% in 90 minutes, then solvent B from 50% to 90% B in 7 minutes and held at 90% for 5 minutes, followed by solvent B from 90% to 5% in 1 minute and re-equilibration for 10 minutes. The flow rate on the analytical column was 300 nL/min. Sixteen tandem mass spectra were collected in a data-dependent manner following each survey scan. MS/MS scans were performed using 60 second exclusion for previously sampled peptide peaks. Data were searched by MaxQuant v1.2.2.5 using the UniProt human database (downloaded 06/2014) [80, 81]. Carbamidomethylation of cysteine, and oxidation of methionine were selected as variable modifications.

### Bioinformatics analysis

Data was imported into Galaxy [82, 83] (http://apostl.moffitt.org/) and analyzed using APOSTL as previously described [34, 84–86]. Briefly, dasatinib enrichments were used as controls for SAINTexpress modeling. A SaintScore cutoff of 0.8 was used to filter for significantly enriched bosutinib specific kinases.

Bosutinib specific kinases were imported into STRING (https://string-db.org/) to determine a potential protein-protein interaction network [39, 40]. The analysis was performed on March 30^th^, 2016 and then confirmed the map on May 17^th^, 2017, which is represented in Figure 2D.

## VI-CELL

Cells were seeded in a 6-well plate (Sarstedt, Nümbrecht, Germany), and treated 24 hours later with vehicle (DMSO) or drug. After treating for 72 hours, the cells were collected and counted using the Vi-CELL XR (Beckman Coulter Life Sciences, Indianapolis, IN). The cell numbers were normalized to the DMSO control which was set to 100%. The experiment was performed in triplicate, and a student's t-test was performed to determine if there was a significant difference between the DMSO control and the everolimus treatments.

### Immunoblotting

Cells were treated with indicated concentrations and times of inhibitors or DMSO as a vehicle control. Cells were harvested in NP40 lysis buffer containing 0.1% NP40, 20 mmol/L Tris HCl (pH 8.0), 0.137 mmol/L NaCl, 10% glycerol, with 1x protease inhibitor cocktail (Roche). Protein concentration was determined using the DC protein assay (Bio-Rad, Hercules, CA) and 20 μg of protein was separated using an 8% SDS-PAGE gel. The resolved proteins were then transferred to Immobilon-Fl membranes (EMD Millipore, Darmstadt, Germany), and incubated overnight at 4°C with the indicated antibodies from Cell Signaling (pY416 Src Family Kinase – 2101; total Src – 36D10; pT202/Y204 MAPK – 9101; total MAPK – 9102; pT308 AKT – 9275; pS473 AKT – 9271; total AKT – 9272; p-S235/236 S6 Ribosomal Protein – 4858; pS240/244 S6 Ribosomal Protein – 5364; total S6 Ribosomal Protein – 2371), Invitrogen (pY861 FAK – 44-626G), Millipore (α-tubulin – CP06), Sigma (β-actin – A5441), and BD Biosciences (total FAK – 610087). The antibodies were diluted 1:1000 in TBS Odyssey Blocking Buffer: 20mM Tris, pH 7.4, 138mM NaCl, with 0.1% Tween added (TBST). Blots were incubated with secondary goat anti-mouse or goat anti-rabbit IRDye antibodies (Licor, Lincoln, NE), and proteins were detected using the Odyssey CLx (Licor). Three Western blots were performed for each biological replicate in order to probe for the indicated proteins. The following antibodies were run on each gel: Gel 1: pY861 FAK, total FAK, pY416 Src, pT202/Y204 ERK, total ERK, and α-tubulin as the loading control, Gel 2: pT308 AKT, total AKT, pS235/236 S6, total S6, and β-actin as the loading control, Gel 3: pS473 AKT, total AKT, pS240/244 S6, total S6, and β-actin as the loading control. After imaging gel 1 on the Odyssey, Gel 1 was stripped and re-probed for total Src. All proteins were normalized to their respective loading control on the same gel (α-tubulin or β-actin). The experiments were performed in triplicate, and a student's t-test was performed to determine if there was a significant difference in protein levels between the DMSO control (set to 1) and the kinase inhibitor treatments.

### Cell apoptotic assay

Cells were plated in 96-well plates in 5% FBS RPMI media on day 1. Twenty four hours later, the media was replaced with 0.1% FBS RPMI media. After 6 hours of starvation, the cells were treated with drug or DMSO as a control. Caspase-Glo 3/7 Assay (Promega, Madison, WI) was used to measure cleaved caspase 3/7 to assess apoptosis 24 hours post treatment. In short, the Caspase-Glo 3/7 reagent was mixed 1:1 ratio with 0.1% FBS RPMI media and then added to the wells. After incubating for 30 minutes, the luminescence is read for each well using a SynergyH1 hybrid plate reader (Biotek). The luminescence signal for each treatment was normalized to the DMSO control (set to 1), and then a t-test was performed to determine if any of the treatments significantly increased caspase 3/7 cleavage compared to DMSO. Three independent experiments were performed and a student's t-test was used to determine significant differences between groups using GraphPad Prism 7.

### Clonogenic assay

Cells were seeded 1000 cells/well in 6 well plates and treated with indicated inhibitors 24 hours later. After 72 hours, the media and drug were replenished and allowed to incubate for another 72 hours. After 6 days, the cells were released from treatment for 1 week, changing the media every 3 days. After the treatment, the cells were washed 2 times with PBS and then fixed with ice cold methanol for 10 minutes. After fixing, the cells were stained with crystal violet (25% methanol, 0.5% crystal violet powder) for 10 minutes and then washed with water to remove the excess stain. The remaining crystal violet stain was measured using the Odyssey CLx 700 channel. The background signal was removed from each reading plate and the staining intensity of each well was normalized to the DMSO control signal (set to 1). Three independent experiments were performed and a student's t-test was used to determine significant differences between groups using GraphPad Prism 7.

### Statistical analysis

Statistical analysis was conducted using GraphPad Prism Version 7 (GraphPad Software, La Jolla, CA). A two-tailed student's t-test was used to determine if there were significant differences between DMSO control and kinase inhibitor treatment in the immunoblot, Caspase-Glo 3/7, Vi-CELL and clonogenic assays. IC50 values from SRB growth assays were calculated using the nonlinear regression analysis with a variable slope in GraphPad Prism 7. Statistical significance was set at p < 0.05 for all experiments (p ^*^ = 0.05 – 0.01, φ = 0.01 – 0.001, δ = 0.001 – 0.001, Ψ < 0.0001).

## SUPPLEMENTARY MATERIALS FIGURES AND TABLES











## Abbreviations

DasRes: dasatinib-resistant
CCCP: compound centric chemical proteomics
BSKs: bosutinib specific kinases
MAPK: mitogen activated protein kinase
