# Supplementary material for: Sustained activation of the AKT/mTOR and MAP kinase pathways mediate resistance to the Src inhibitor, dasatinib, in thyroid cancer
Source: Oncotarget. 2017 Aug 24;8(61):103014–31. doi: 10.18632/oncotarget.20488 (PMC5732707; doi:10.18632/oncotarget.20488)
Supplement: Supplementary file 1 [file oncotarget-08-103014-s001.pdf]

Sustained activation of the AKT/mTOR and MAP kinase pathways mediate resistance to the Src inhibitor, dasatinib, in thyroid cancer

SUPPLEMENTARY MATERIALS

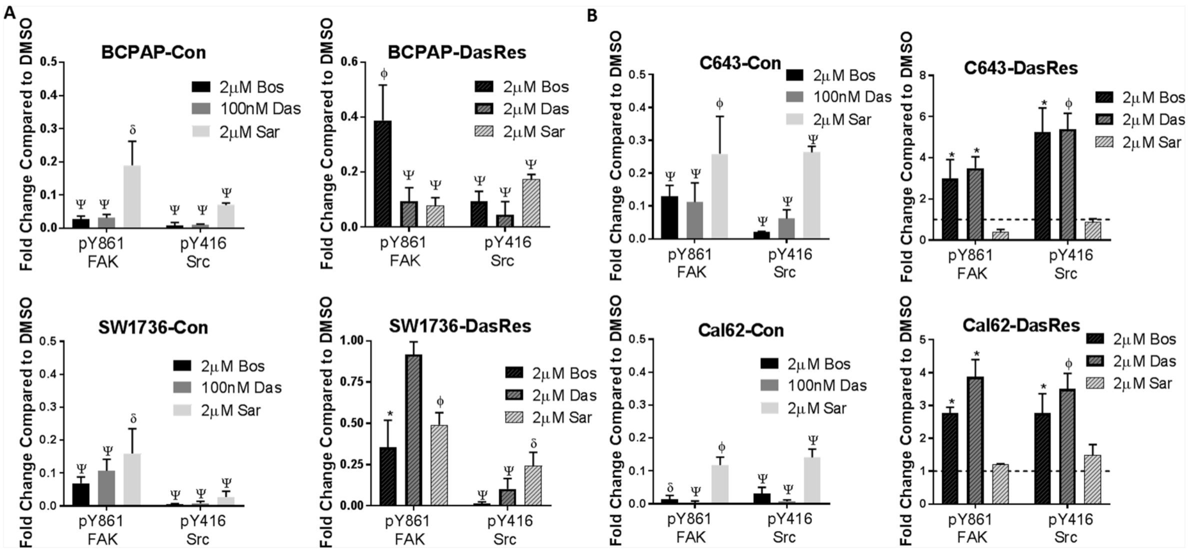

Supplementary Figure 1: Dasatinib resistant cells exhibit differential sensitivity to the Src inhibitors, saracatinib and bosutinib. (A-B) Quantification of pY861-FAK and pY416-Src western blot after Src inhibitor treatment for 24 hours in the (A) BRAF-mutant and (B) RAS-mutant control and DasRes cell lines. Signal intensity was determined using Image Studio 4.0. Each protein was normalized to the respective loading control (α-tubulin for pY861-FAK, total FAK, pY416-Src, and total Src), and then fold changes were calculated by setting the DMSO signal to 1. Three independent biological replicates were performed, and the standard error mean is displayed in the quantification graphs. p-value * = 0.05 – 0.01, φ = 0.01 – 0.001, δ = 0.001 – 0.001, Ψ < 0.0001.

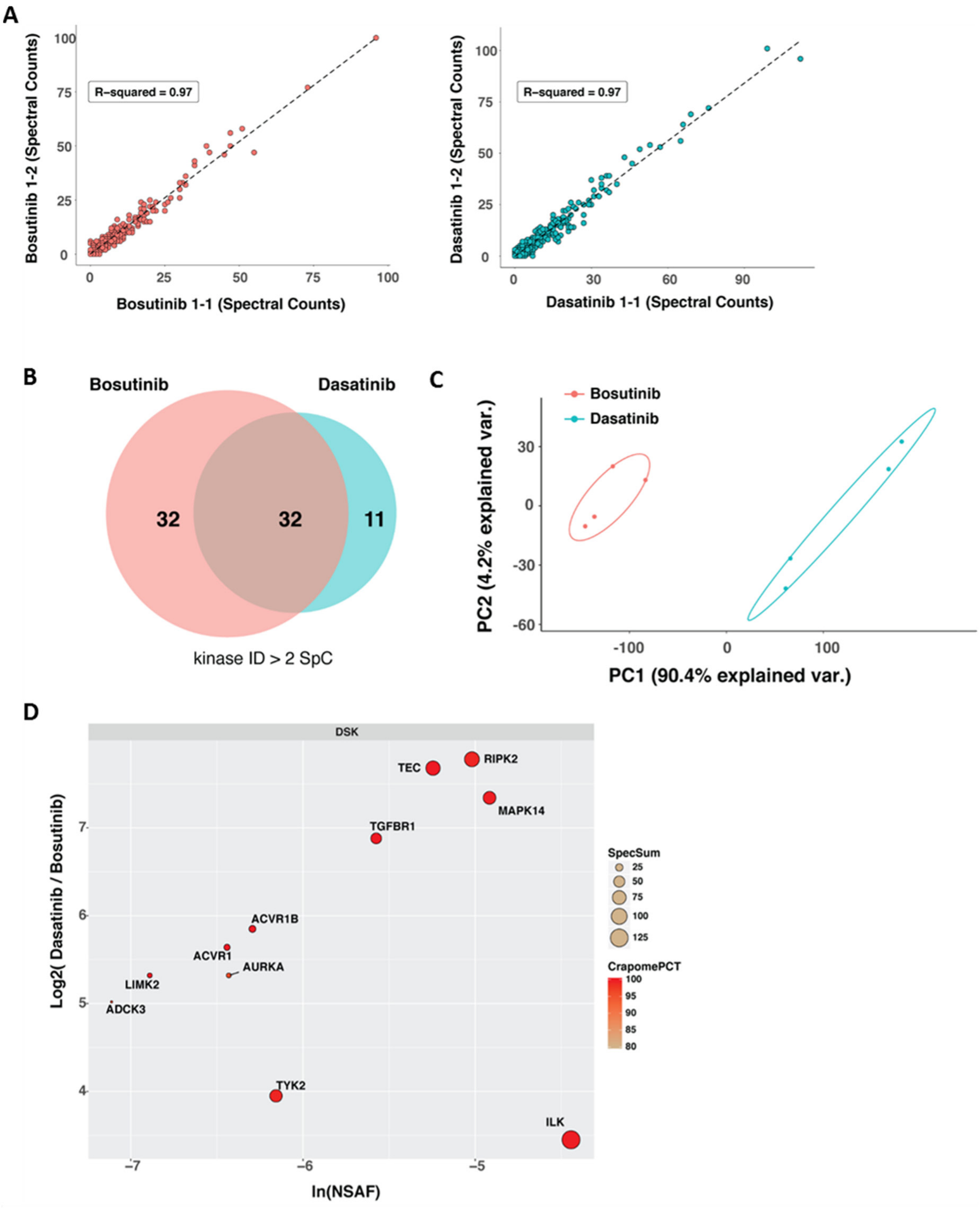

Supplementary Figure 2: Chemical proteomic characterization of bosutinib and dasatinib in BCPAP cells. (A) Correlations between technical replicates of bosutinib and dasatinib pulldowns. **(B)** Venn Diagram comparing number of kinases identified in bosutinib and dasatinib pulldowns with >=2 spectral counts. **(C)** Principal component analysis comparing complete chemical proteomic profile of bosutinib and dasatinib. **(D)** Protein kinase interaction profile of dasatinib in BCPAP-DasRes cells as determined by NSAF and ratio of spectral counts relative to dasatinib. NSAF: normalized spectral abundance factor; CRAPomePCT: percent probability of specific interaction based on CRAPome database. Displayed are kinases with SaintScore >= 0.8.

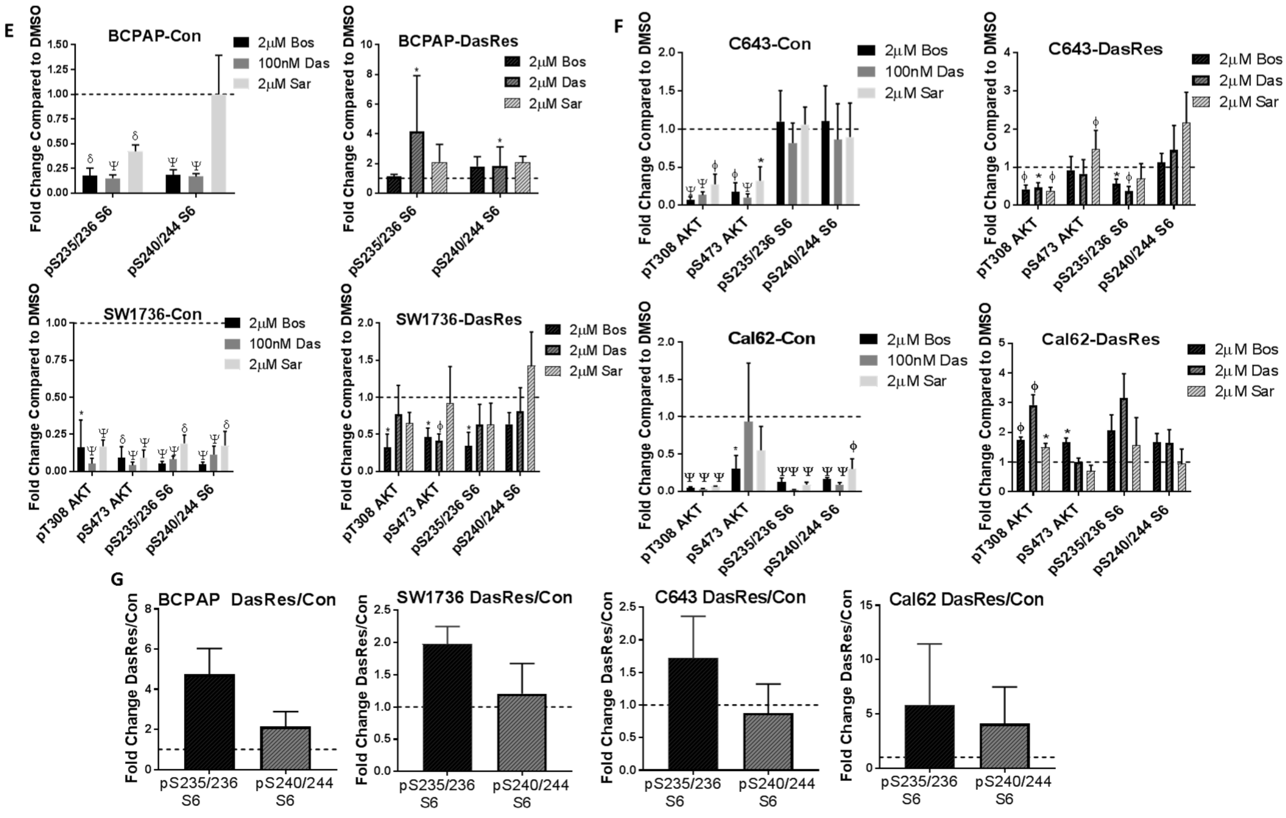

Supplementary Figure 2 (*Continued*): (E-F) Quantification of downstream targets of the AKT/mTOR (AKT, S6) and MEK (ERK) pathways were assessed via Western blot after Src inhibitor treatment for 24 hours in the (E) BRAF-mutant and (F) RAS-mutant control and DasRes cell lines. Signal intensity was determined using Image Studio 4.0. Each protein was normalized to the respective loading control (α-tubulin for pT202/pY204-ERK, total ERK; β-actin for pT308-AKT, total AKT, pS235/236 S6, total S6 (run on one gel) and pS473-AKT, total AKT, pS240/244 S6, and total S6 (run on one gel)) and fold changes were calculated by setting the DMSO signal to 1. Three independent biological replicates were performed, and the standard error mean is displayed in the quantification graphs. p-value * = 0.05 – 0.01, φ = 0.01 – 0.001, δ = 0.001 – 0.001, Ψ < 0.0001. **(G)** Baseline phosphorylation of S6 was determined by comparing DMSO treated control and DasRes cell lines via Western blotting in Figure 2E and 2F. Average signal was calculated from three independent experiments, and then the DasRes signal was compared to the respective control cell line to determine the fold change in baseline pathway activity. Three independent biological replicates were performed, and the standard error mean is displayed in the quantification graphs.

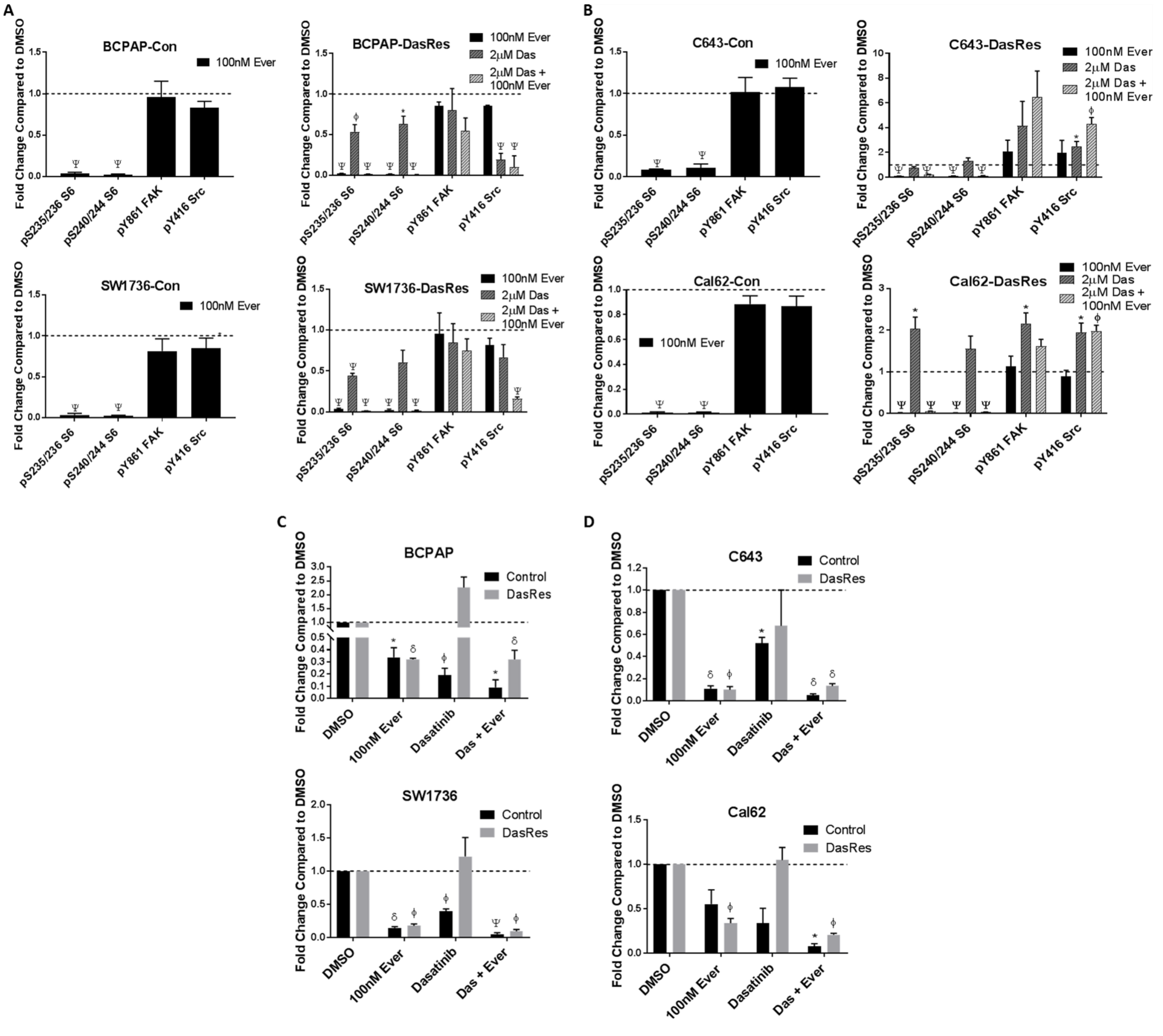

Supplementary Figure 3: The mTOR inhibitor, everolimus, inhibits cell growth and clonogenicity, but does not induce apoptosis. (A-B) Quantification of downstream targets of the AKT/mTOR (S6) pathway was assessed via Western blot after Src inhibitor treatment for 24 hours in the (A) BRAF-mutant and (B) RAS-mutant control and DasRes cell lines. Signal intensity was determined using Image Studio 4.0. Each protein was normalized to the respective loading control (α-tubulin for pY861-FAK, total FAK, pY416-Src, total Src; β-actin for pS235/236 S6, total S6 (run on one gel); β-actin for pS240/244 S6, and total S6 (run on one gel) and fold changes were calculated by setting the DMSO signal to 1. Three independent biological replicates were performed, and the standard error mean is displayed in the quantification graphs. p-value * = 0.05 – 0.01, φ = 0.01 – 0.001, δ = 0.001 – 0.001, Ψ < 0.0001. **(C-D)** Quantification of the (C) BRAF-mutant and (D) RAS-mutant clonogenic assay. Crystal violet intensity was measured using the Odyssey CLx 700 channel and Image Studio 4.0 software. Background was subtracted from the signal intensity and fold changes were calculated by normalizing the signal intensity of the treatments to DMSO. Control cells were treated with 100nM dasatinib and the DasRes cells were treated with 2μM dasatinib. Three independent biological replicates were performed, and the standard error mean is displayed in the quantification graphs. p-value * = 0.05 – 0.01, φ = 0.01 – 0.001, δ = 0.001 – 0.001, Ψ < 0.0001.

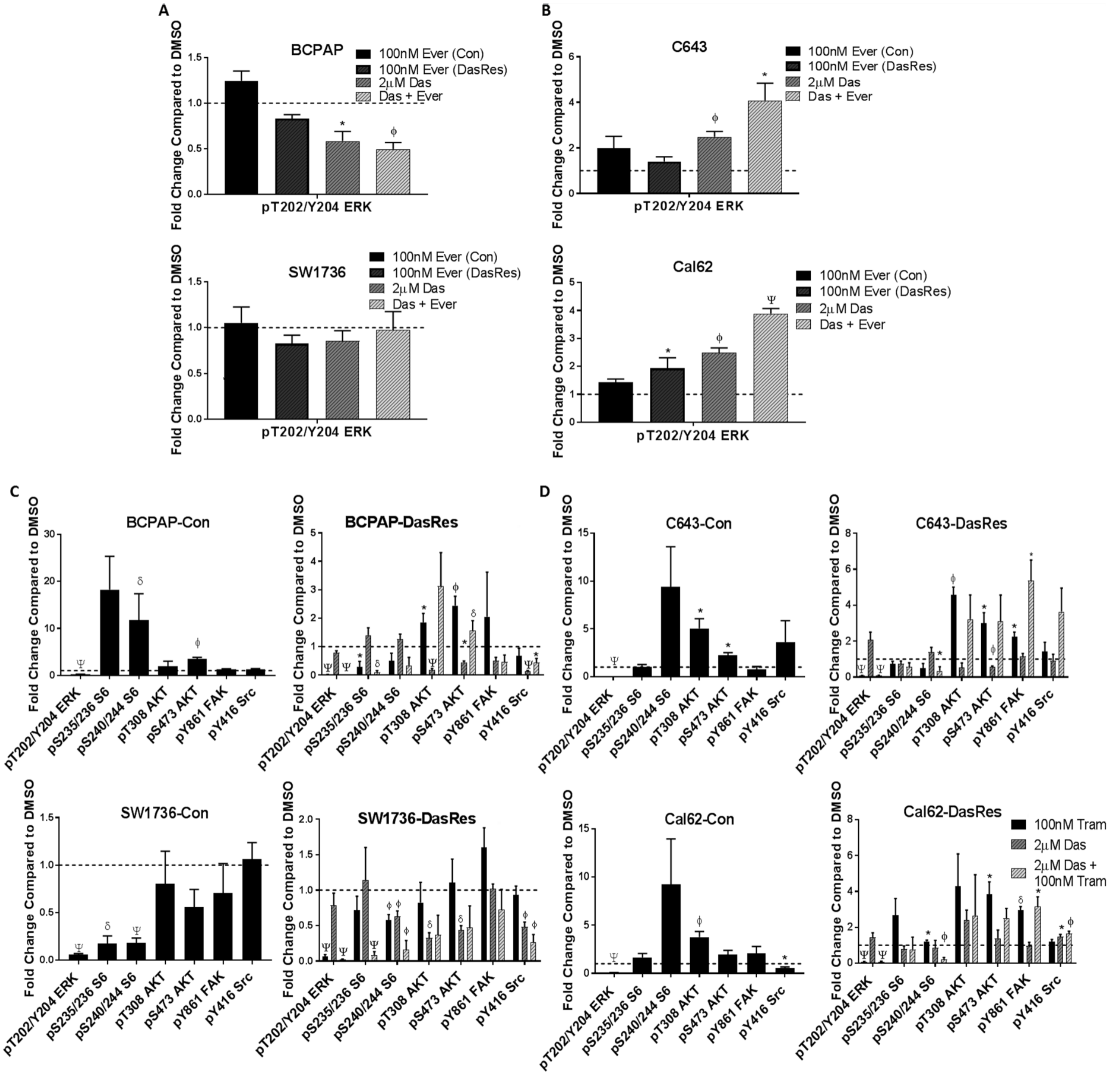

Supplementary Figure 4: Dasatinib-resistant cells are able to switch between PI3K/AKT and MAPK pathways in response to inhibitor treatment. (A-B) Quantification of downstream targets of the MAPK pathway (ERK) was assessed via Western blot after everolimus treatment for 24 hours in the (A) BRAF-mutant and (B) RAS-mutant control and DasRes cell lines. Signal intensity was determined using Image Studio 4.0. Each protein was normalized to the respective loading control and fold changes were calculated by setting the DMSO signal to 1. Three independent biological replicates were performed, and the standard error mean is displayed in the quantification graphs. p-value * = 0.05 – 0.01, φ = 0.01 – 0.001, δ = 0.001 – 0.001, Ψ < 0.0001. **(C-D)** Quantification of downstream targets of the MAPK (ERK) and AKT/mTOR (S6) pathways were assessed via western blot after everolimus treatment for 24 hours in the (C) BRAF-mutant and (D) RAS-mutant clonogenic assay. Signal intensity was determined using Image Studio 4.0. Each protein was normalized to loading control (α-tubulin for pY861-FAK, total FAK, pY416-Src, total Src, pT202/204-ERK, total ERK; β-actin for pT308-AKT, total AKT, pS235/236-S6, total S6 (run on one gel); β-actin for pS473-AKT, total AKT, pS240/244-S6, total S6 (run on one gel)) and fold changes were calculated by setting the DMSO signal to 1. Control cells were treated with 100nM dasatinib and the DasRes cells were treated with 2μM dasatinib. Three independent biological replicates were performed, and the standard error mean is displayed in the quantification graphs. p-value * = 0.05 – 0.01, φ = 0.01 – 0.001, δ = 0.001 – 0.001, Ψ < 0.0001.

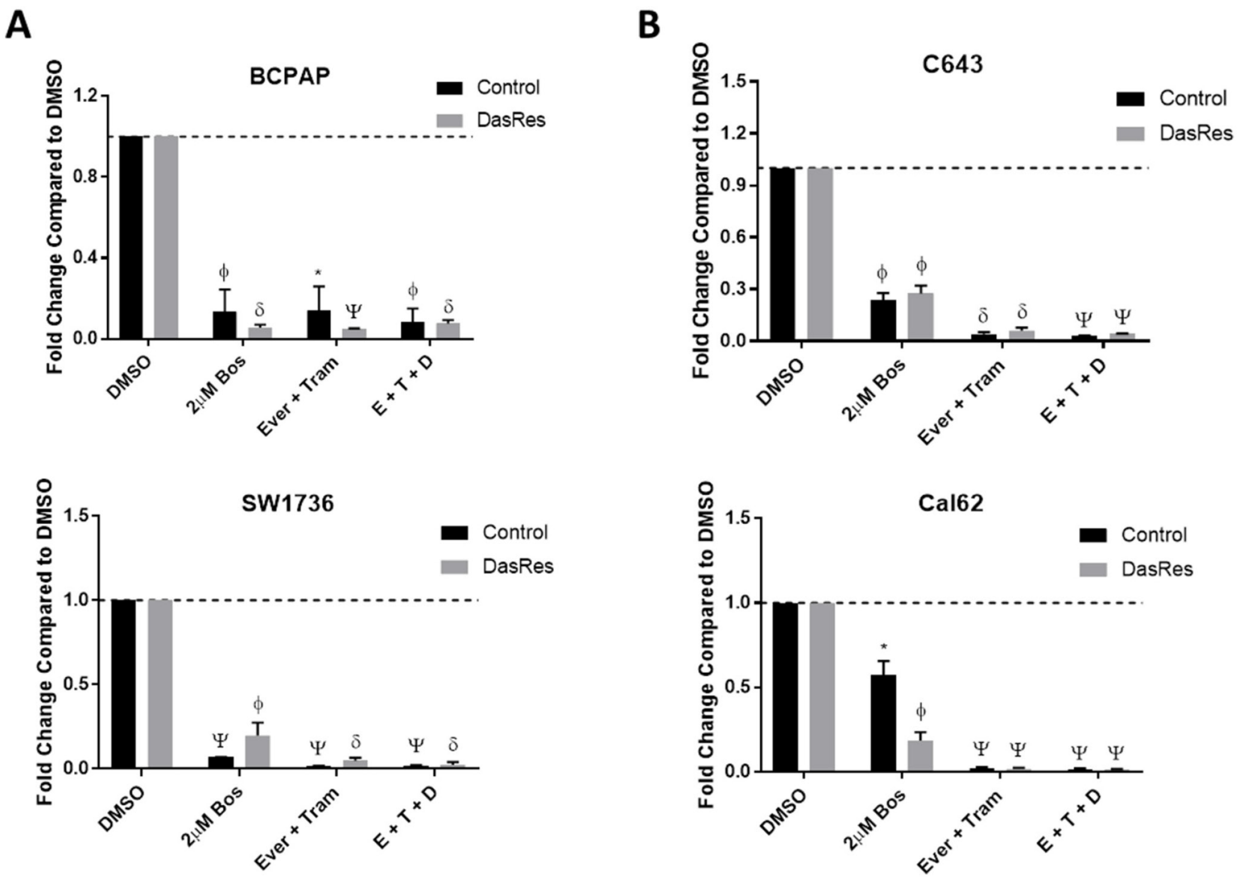

Supplementary Figure 5: Dasatinib-resistant cells are able to utilize both PI3K/AKT and MAPK pathways to survive single agent therapy. (A-B) Quantification of the (A) BRAF-mutant and (B) RAS-mutant clonogenic assays. Crystal violet intensity was measured using the Odyssey CLx 700 channel and Image Studio 4.0 software. Background was subtracted from the signal intensity and fold changes were calculated by normalizing the signal intensity of the treatments to DMSO. Control cells were treated with 100nM dasatinib and the DasRes cells were treated with 2μM dasatinib. Three independent biological replicates were performed, and the standard error mean is displayed in the quantification graphs. p-value * = 0.05 – 0.01, φ = 0.01 – 0.001, δ = 0.001 – 0.001, Ψ < 0.0001.

Supplementary Table 1: Chemical proteomics data (Spectral Counts). Data was searched by Mascot and displayed are unique spectrum counts. A minimum of 2 unique spectra were required for protein ID. Data was filtered with 95% protein and peptide cutoffs. CT: competition.

See Supplementary File 1

Supplementary Table 2: Dasatinib and bosutinib *in vitro* kinase assays. An *in vitro* kinase assay was performed to determine the percent inhibition of each kinase identified in the pull down at 500nM, 1µM, or 10µM concentrations of either dasatinib or bosutinib. Kd values were also calculated. Red boxes indicate percent activity was not detected.

See Supplementary File 2

Supplementary Table 3: Bosutinib specific interactions identified from SAINTexpress and APOSTL. Unfiltered results from SAINTexpress and APOSTL analysis.

See Supplementary File 3

Supplementary Table 4: STRING interactions. Categories indicating how protein-protein interactions were determined.

See Supplementary File 4
